# Design of Edge-IoMT Network Architecture with Weight-Based Scheduling

**DOI:** 10.3390/s23208553

**Published:** 2023-10-18

**Authors:** Li-Min Tseng, Ping-Feng Chen, Chih-Yu Wen

**Affiliations:** 1 Department of Electrical Engineering, National Chung Hsing University, Taichung 402, Taiwan; liminzz121@gmail.com (L.-M.T.); harrychen@smail.nchu.edu.tw (P.-F.C.); 2 Smart Sustainable New Agriculture Research Center (SMARTer), National Chung Hsing University, Taichung 402, Taiwan; 3 Innovation and Development Center of Sustainable Agriculture (IDCSA), National Chung Hsing University, Taichung 402, Taiwan

**Keywords:** clustering, topology management, data compression, scheduling algorithm, network traffic control

## Abstract

Population health monitoring based on the Internet of Medical Things (IoMT) is becoming an important application trend healthcare improvement. This work aims to develop an autonomous network architecture, collecting sensor data with a cluster topology, forwarding information through relay nodes, and applying edge computing and transmission scheduling for network scalability and operational efficiency. The proposed distributed network architecture incorporates data compression technologies and effective scheduling algorithms for handling the transmission scheduling of various physiological signals. Compared to existing scheduling mechanisms, the experimental results depict the network performance and show that in analyzing the delay and jitter, the proposed WFQ-based algorithms have reduced the delay and jitter ratio by about 40% and 19.47% compared to LLQ with priority queueing scheme, respectively. The experimental results also demonstrate that the proposed network topology is more effective than the direct path transmission approach in terms of energy consumption, which suggests that the proposed network architecture may improve the development of medical applications with body area networks such that the goal of self-organizing population health monitoring can be achieved.

## 1. Introduction

Considering the current risk of pandemics, real-time demand for multi-access medical monitoring, information mining, and effective disease diagnosis, group health monitoring implemented with the Internet of Medical Things (IoMT) will be an important application trend for improving health care in the future. Due to the extensive use of wireless communication technologies, the Internet of Things can seamlessly and transparently incorporate more heterogeneous smart devices or end devices. However, for IoMT applications that require higher accuracy, real-time response, and robustness, cloud-based architectures may cause adverse effects in case of network failure or insufficient bandwidth, which may lead to medical emergencies. Therefore, by collecting and analyzing data at the network edge, we may improve network performance and reliability (e.g., with greater operation flexibility, lower latency, energy consumption, and location awareness) for decentralized healthcare applications.

With the development of medical embedded systems and telemedicine technologies, the demands for storing and transmitting physiological signals are increasing. However, the process of real-time monitoring or group monitoring leads to the generation of large amounts of physiological signals. One solution to deal with this scenario is the use of data compression techniques, which conserves signal storage space and facilitates transmission, reception, and processing of the data. Currently, many effective data compression methods for physiological signals are widely used. Data compression methods are classified into three types [1]: (a) direct data compression method, (b) transformational data compression methods, and (c) parameter extraction-based data compression methods [2]. Among these three categories, transform based data compression is always preferred, because these techniques provide efficient results in terms of CR and signal reconstruction [3]. In these categories, the signal is first transformed from one domain (mostly time domain) to the other domain and then rejection of irrelevant coefficients is performed. The examples of transformed-based techniques are: sine transform, Fourier transform (FT), cosine transform, wavelet transform (WT), etc. Since the wavelet transform is useful to locate and analyze the non-stationary physiological signals (e.g., electrocardiography (ECG), electroencephalography (EEG), and electromyography (EMG)) in both time and frequency domains, it is applied for data compression of physiological signals in this work.

Data gathering is a critical process, which includes protocols and scheduling strategies to fulfill the network requirements (e.g., increased lifetime, reliability, and self-configuration). Scheduling is a process by which the system determines which task should be executed at the given time, and thus achieves load balancing and efficient system resource allocation. However, improper scheduling algorithms may waste transmission resources or lead to performance degradation (e.g., packet collisions). To organize the time constraints of multiple data forwarding tasks, these tasks need to be carefully arranged to adjust the load well between sensors. Many queue scheduling algorithms have been proposed, where weighted fair queueing (WFQ) is one of the widely used scheduling algorithms due to its fair bandwidth allocation and traffic prioritization capabilities.

Besides considering the scheduling schemes, to conserve limited energy resources, aggregate information from individual sensor nodes, and further provide network scalability and robustness, in this paper, sensor nodes are grouped into clusters, where network topology management is performed by setting weights and countdown timers between sensors, clusterhead (CH), inter-clusterhead (inter-CH), relay and base station through a hierarchical clustering approach. The data size, priority, and transmission scheduling of various physiological signals are also considered, the data are compressed at the network edge and the transmission bandwidth of each physiological signal is assigned through a scheduling algorithm. The proposed method ensures data transmission frequency and quality of service for different types of physiological signals, as well as simplifies data transmission traffic and improves data transmission efficiency.

The proposed scheduling method can simplify the data transmission traffic and increase the data transmission efficiency. The major contributions and key features of this paper are: (1) explore the architecture of the group health monitoring system on basis of the relay-based wireless body area network (WBAN); (2) address routing and relay selection schemes for the proposed architecture; (3) devise the specific health monitoring architecture based on the IoMT, considering signal prioritization and transmission scheduling through the hierarchical clustering method to organize sensors by setting weights, and applying data compression techniques at the network edge.

The rest of the paper is organized as follows: Section 2 discusses the related works. Section 3 explains the functionality and operation of the proposed clustering methods, compression technique, and scheduling algorithms. Section 4 presents the experiment scenario and analyzes the experiment results. Section 5 draws conclusions and future work.

## 2. Related Works

This section briefly describes the existing works about edge-based IoMT applications, network topology control, and queueing management.

### 2.1. Edge-Based IoMT

The IoMT is expected to enable cost-effective application in the healthcare field, especially for effective monitoring of dependent or chronic patients [4]. The combination of IoT and Deep Learning (DL) enables highly effective health monitoring systems. For instance, the health information generated by IoT devices are preprocessed and classified at edge computing and data transmission to the cloud server [5], or even primary disease prediction and patient life extension at home [6].

**Figure 1:**
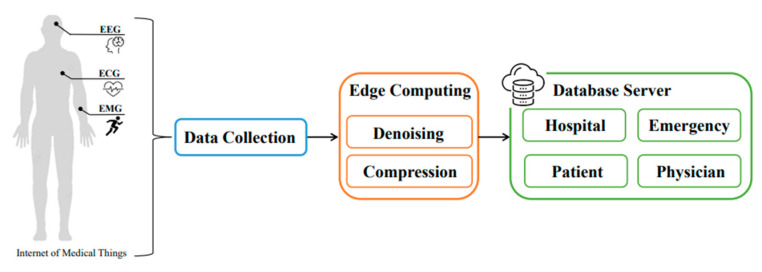
The overall architecture for edge computing enabled IoMT systems (modified and reproduced from [5]).

Edge computing is considered as local processing, where the data are computed closer to the network edge than where the data are generated. Edge computing optimizes cloud computing systems by processing data at the edge of distributed networks and effectively reduces the amount of data that must be transferred, reducing the network traffic and the distance of data transmission [7]. For large-scale IoT edge computing, considering real-time, reliability, and accuracy, lightweight trust algorithms are more reliable for dealing with the attacks [8]. Accordingly, as the edge devices can spontaneously identify the data, it is an important target for healthcare applications in edge-based IoMT [9]. Figure 1 shows a conceptional edge-IoMT network architecture.

### 2.2. Network Topology Control

Wireless Sensor Networks (WSNs) typically include a large number of sensors that collect and transmit data to remote devices for subsequent processing. Although WSNs are highly scalable networks, network management has been an issue given the scale of deployment and associated quality issues such as resource management, robustness, and scalability. Topology management is considered as a possible technique to address these issues. Referring to [10], the authors proposed a two-level topology design approach. The data from each sensor are transmitted to the base station through a predefined CH. There are only two hops from the sensor to the base station, which increases the packet delivery and extends the lifetime of the network.

There are several existing studies considering network topology control schemes. For instance, selecting a CH by distance information or residual energy to improve load balancing, energy consumption, and quality of service [11]. Due to less efficient management and collisions, there is a waste of unused time slots and bandwidth. Thus, Ambigavathi [12] proposes an efficient traffic priority-based channel access technique to reduce the transmission delay of critical packets and to address the problem of collision with priority nodes. The different models and experimental results are compared and contrasted in [13], where the network behaviors are explored by physical channel modeling, propagation, and link layer performance.

### 2.3. Scheduling on IoMT

This subsection briefly describes the latest scheduling techniques for IoMT applications. Ullah et al. [14] propose traffic priority-based delay-aware and energy-efficient path allocation routing protocol for wireless body area network, which selects the optimal paths with high residual energy of nodes with minimum temperature rise. An energy-efficient and delay-aware path allocation algorithm is developed for normal data, performing the selection of optimal and shortest paths. Then, a data on-demand algorithm is derived for on-demand data dissemination. Finally, criticalities detection algorithms are developed for measuring criticalities of vital signs and allocation of adaptive and energy-efficient paths on the priority basis. Yazdi et al. [15] propose a priority-based MAC protocol for transmitting normal data, periodic data, and emergency data in wireless body area networks. This MAC design aims to guarantee energy consumption and delay reduction with traffic prioritization and classification for achieving the desired performance.

To avoid data losses and reduce dissemination time with the priority-based data, Alsiddiky, et al. [16] present the selective decision modes (i.e., congestion mitigation and perfect queuing modes) for achieving successful data delivery. Depending on the rate of congestion and priority of the data sensed, transmission is balanced using queuing or delivery as modeled in the decision mode. For real-time remote monitoring of the patient, Elshahed et al. [17] propose a new task scheduling and allocation technique for healthcare monitoring implemented in IoT cloud-based architecture. The proposed technique performs a prioritized sorted task-based allocation scheme for selecting the best virtual machine to execute the health task, solving the problem of delay, missed tasks, and failure rate, and the guarantee ratio.

Besides the notable scheduling techniques, the genetic algorithm (GA), a metaheuristic approach that deals with the foundations of hereditary qualities and regular determination, is applied for scheduling in IoMT applications. Hussain [18] puts forth the significance of scheduling management and the application of AI in the IoMT-cloud environment. Singh et al. [19] explore the energy utilization of the IoMT with the GA evolutionary processing based on the dynamic sensor range, introducing multiple sinks and direct information collection concepts, which provide a robust approach for massive data collection.

### 2.4. Comparative Analysis of Queueing Managements

In WSNs, data transfer from the source node to the destination is carried out through several intermediate nodes, so each node may have multiple data packets stored in it. However, depending on the application requirement, it can be categorized into several types of packets (e.g., real-time or non-real-time transmissions). Justu [20] proposes a data scheduling technique based on latency and loss constraint, where the input data are prioritized with latency and loss constraint conditions, and data with high priority are scheduled based on data priority, which guarantees that high-priority data are delivered instantly. The increasing number of real-time applications makes the requirements for network traffic and bandwidth management critical. Bahaweres [21] describes a fair queuing with the scheduling of Weighted Fair Queuing (WFQ) and Low Latency Queueing (LLQ), which divides the available bandwidth across queues of traffic based on weights to ensure that the important traffic receives a higher priority than the less important traffic.

To evaluate the performance of queueing management, several parameters (e.g., the amount of traffic, type of traffic, services with mixed traffic, and the number of queues on the router) are analyzed for the following queuing mechanisms [22]: First-In First-Out (FIFO), Custom Queuing (CQ), Priority Queuing (PQ), Weighted Fair Queuing (WFQ), Class Based Weighted Fair Queuing (CBWFQ), and Low Latency Queuing (LLQ). The study of [23] investigates the following scheduling algorithms: Round Robin (RR), Strict Priority (SP), Weighted Fair (WF), and Weighted Round Robin (WRR). Note that each queuing algorithm has its advantages when handling certain types of traffic. Network performance metrics (i.e., throughput and endpoint latency) and queuing metrics (i.e., peak queue size, average queue length, average queue time, and total packet loss) can be applied to evaluate the performance of scheduling algorithms.

Moreover, given the growth and demand for smart networks and intelligent devices, Quality of Service (QoS) is considered as an important parameter to support data quality in IoT. Three queuing algorithms such as FIFO, PQ, and WFQ are analyzed and compared by using a QoS mechanism to control and observe the congestion in the IoT network [24]. A scalable load business flow model is performed and analyzed in [25]. The algorithm can be used to flexibly adjust the queue weights according to the real-time metrics monitored by the network to ensure the quality of service of various disaster recovery services. Table 1 and Table 2 summarize the characteristics, features, strengths, and weaknesses of the above-mentioned queueing algorithms.

## 3. System Architecture

This section presents the proposed IoMT architecture, including data collection and preprocessing schemes, a priority-based topology design approach, and scheduling algorithms for physiological signals.

### 3.1. IoMT Architecture

As the demand for health monitoring increases, this work focuses on the design of network architecture and data analysis techniques for the IoMT system, consisting of sensor nodes, relay nodes, and the base station. Through priority-based clustering, weight assignment, data compression, and scheduling algorithms, the performance of group monitoring is examined in terms of transmission load and latency.

**Figure 2:**
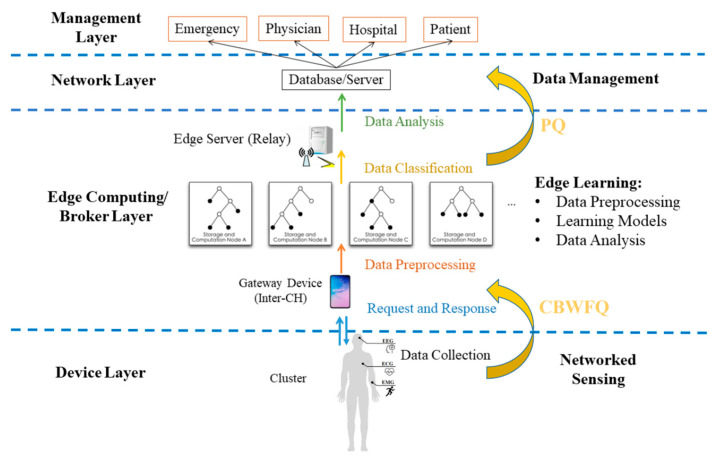
A four-layer IoMT architecture.

Most of the current IoMT systems are divided into four layers, as shown in Figure 2, starting from personal physiological signal collection to analysis and ending in data storage and management. Observe that the first architecture component is the device layer, which uses wearable sensors to collect physiological signals from patients. The patients are grouped into clusters based on disease severity. Then, the wearable device transmits the data to the second architecture component, the broker layer, over wireless communication protocols such as Bluetooth or Wi-Fi. Due to the processing and storage limitations of the device, the members in the cluster first transmit the compressed physiological signals to local controllers (i.e., a CH or an inter-CH), and then the more powerful relay node performs decompression and data analysis. The details of compression and clustering algorithms are discussed in the following subsections, respectively. The third architecture component is the network layer, where the relay node sends the data to the database through the network after decompression and data analysis. The last architecture component is the management layer. Through the collected and analyzed data, health care professionals can monitor the physical conditions of all patients and handle the emergencies.

### 3.2. Data Collection

This subsection describes the dataset and the procedures of data preprocessing. Note that this work does not consider the handling of data security issues in data collection, data scheduling, and data compression. A brief analysis of data compression security issues and implementing efficient data compression and encryption schemes in network services can be found in [27,28,29].

#### 3.2.1. Dataset Description

For healthcare professionals and patients, the long-term monitoring of physiological signals is beneficial to the rational assessment of health status and disease evolution. As we know, physiological signals reflect the electrical activity of a specific body part and provide valuable information about mood, cognition, and many other health issues, thus any deviation from the norm in patterns may indicate an underlying health problem. For instance, arrhythmias can be picked up by ECG signals while epilepsy, which is a common neurological disorder, manifests itself as abnormalities in EEG signals during epileptic seizure. Monitoring and analyzing these physiological signals form the basis of biomedical devices used for the diagnosis, detection, and treatment of various diseases [30]. Moreover, in recent years, stress and mental health have been considered as important worldwide concerns. Stress detection using physiological signals such as ECG, EMG, and EEG is a traditional approach [31]. Therefore, three critical physiological signals are selected, namely ECG, EEG, and EMG.

- ECG: Since cardiovascular diseases may lead to sudden cardiac arrest or even death and are difficult for patients to detect and handle them effectively in time, it is necessary to monitor the ECG signal in real-time to prevent this situation. Here, we apply the MIT-BIH dataset as one of the input data, which is publicly available at PhysioNet [32]. The dataset provides data collected from 48 patients with recordings of 30 min each. The recordings are digitized at 360 samples per second per channel with 11-bit resolution over a 10 mV range.
- EEG: Many studies have shown that patients with head trauma or cerebrovascular disease can be managed effectively with continuous EEG monitoring, and there is also a lot of usage of EEG data analysis for effective diagnosis of certain diseases such as epilepsy. This work applies the EEGLAB dataset [33], which uses a single structure to store data, acquisition parameters, events, channel locations, and epoch information. The sample dataset consists of 32 channels, and the sampling frequency is 128 Hz. Channel 1 (FP1) and channel 2 (FP2) in the frontal area are adopted as inputs, which are commonly used for EEG analysis.
- EMG: Depending on the position of the patch, the collected EMG data can be used for different applications. The EMG dataset in bio-signals Repository [34] is used to control the prosthesis movements. The required finger movements are performed through 8 participants with ten categories of individual and combined finger movements (e.g., thumb, index, middle, ring, little, thumb-index, thumb-middle, thumb-ring, thumb-little, and hand-close finger movements). The data were collected using two EMG channels and the sample rate is 450 Hz. In this work, the hand-close dataset is selected as the EMG signal input.

#### 3.2.2. Data Preprocessing

For physiological signals, the signal clarity is extremely important. However, most of these physiological signals are non-stationary or non-linear, and during the data acquisition process, the signals are easily contaminated by noise. Therefore, the signal quality needs to be improved by preprocessing to remove noise to ensure the accuracy of medical analysis and diagnosis. With the characteristics of time and frequency domain analysis, in this work, wavelet transform (WT) is applied to decompose a signal into several scales with various frequency bands. At each scale, the signal’s instantaneous structures can be determined approximately, which are available for denoising.

**Figure 3:**
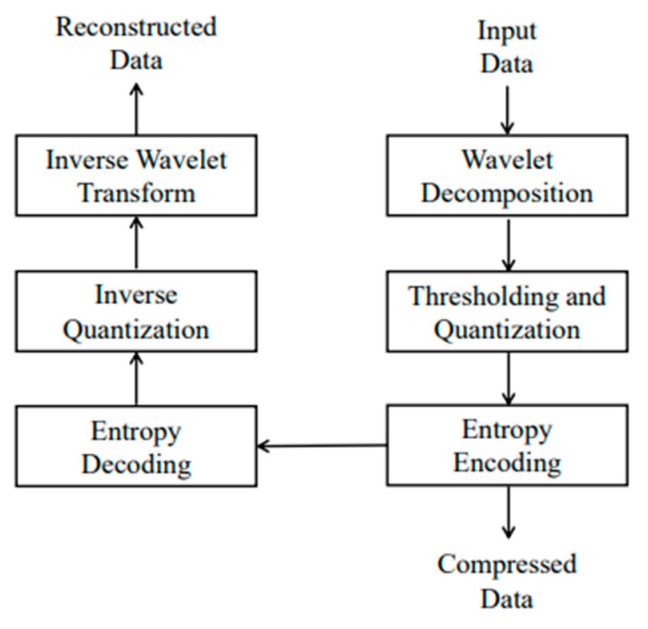
Structure of compression with wavelet transform.

Due to the increased amount of data and transmission power consumption with long-time monitoring, a data compression technique is essential, which achieves effective processing of physiological information, reduces the transmission load, and shortens the communication time. Since most of the physiological signals are non-stationary, the analysis method should not only have good resolution in the frequency domain, but also needs to have to localize signals in time domain. Based on the requirement, Discrete Wavelet Transform (DWT) is a wavelet transformation in which wavelets are discretely sampled, and it captures both frequency and position information of time such that the signal is approximately decomposed with detailed coefficients at each level through a process of low pass filtering, high pass filtering, and soft threshold function, respectively [35,36,37,38,39,40]. For the denoised ECG, the wavelet basis function is db5 wavelet and the decomposition architecture is five-layer; db5 wavelet and three-layer decomposition are used to decompose EEG signals; EMG signals use db6 wavelet and three-layer decomposition for signal decomposition. Figure 3 illustrates the compression structure with WT.

### 3.3. Priority-Based Clustering Scheme

**Figure 4:**
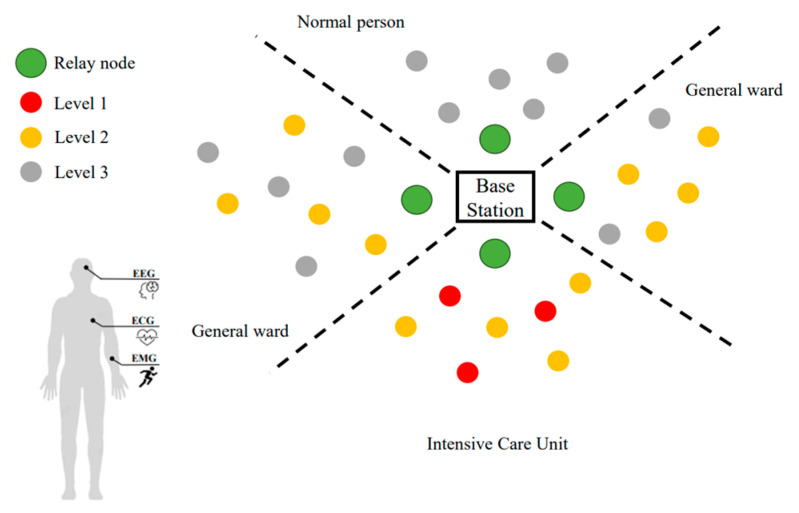
A scenario of patients with different physical signals of priority weights.

In consideration of reducing the demand for medical services by keeping people healthy, monitoring of health parameters is likely to become necessary to solve this problem. In recent years, many sensor networks have been integrated with health monitoring to provide medical staff with a more efficient and extensive management approach, reducing nursing workload and providing patients with a clearer understanding of their current physical status. In this section, we focus on the network structure of group health monitoring in wards. The clustering algorithm is referenced from [41]. The network topology is formed into three phases: (1) Phase 1: clustering and selecting inter-CH, (2) Phase 2: CHs and inter-CHs scheduling and management, and (3) Phase 3: inter-CHs and relay scheduling and management. Figure 4 shows a scenario of our implementation, where there are three levels of patients depending on their severity and it is assumed the relay nodes and base station are fixed.

#### 3.3.1. Phase 1: Clustering and Inter-CH Selection

For establishing a cluster-based network topology, each member scans how many neighboring devices can join the cluster by broadcasting, and then estimates the distances of the nearby devices with the RSSI value obtained during the handshake process. When a member (say node *i*) broadcasts its priority information (i.e., levels 1–3) to other neighboring devices, the countdown timer, *WT_i_*, is triggered. When the device receives a message from a neighboring node and the timer expires, then it sends an address information to other nodes to indicate that it has become a CH, and the adjacent nodes join the cluster at *WT_i_* = 0. 

**Figure 5:**
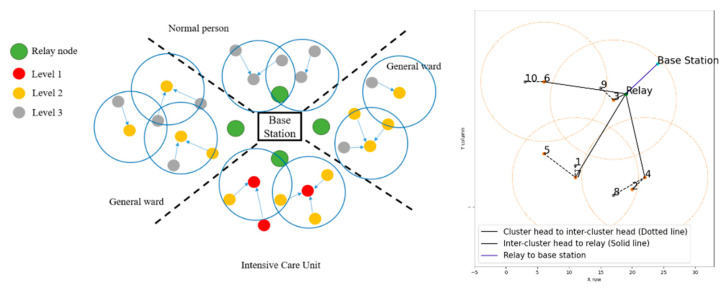
Network clustering and inter-CH selection (**left**); a typical run of network topology (**right**), where a solid line and a dotted line represent the information directions from an inter-CH to a relay node and a CH to an inter-CH, respectively.

Similarly, the adjacent CHs can follow the above procedures to perform the inter-CH selection. Accordingly, based on the neighboring network density and patient’s priority information, we may set the initial random waiting timer from the distribution $\beta \cdot {WT}_{i}^{\left(0\right)}+\alpha$ [42]. The algorithm mentioned above is based on the countdown timer and describes the parameters by priority and RSSI. α is the priority of each member, and we have three levels in this system. For instance, for level 1 (highest level) α = 0 and level 3 (lowest level) α = 2. There is a situation in which multiple patients of the same priority may be in the same ward, so the RSSI value of the device and the relay, β, is a critical parameter of the formula that the one closer to the relay will count down faster. Therefore, according to the different priority levels, higher priority devices will receive a shorter waiting time, which means it is more likely to be the head, because some patients with higher priority are high-risk people that require frequent and real-time monitoring. The conceptual communication architecture after clustering and inter-CH selection are shown in Figure 5 (left).

**Figure 6:**
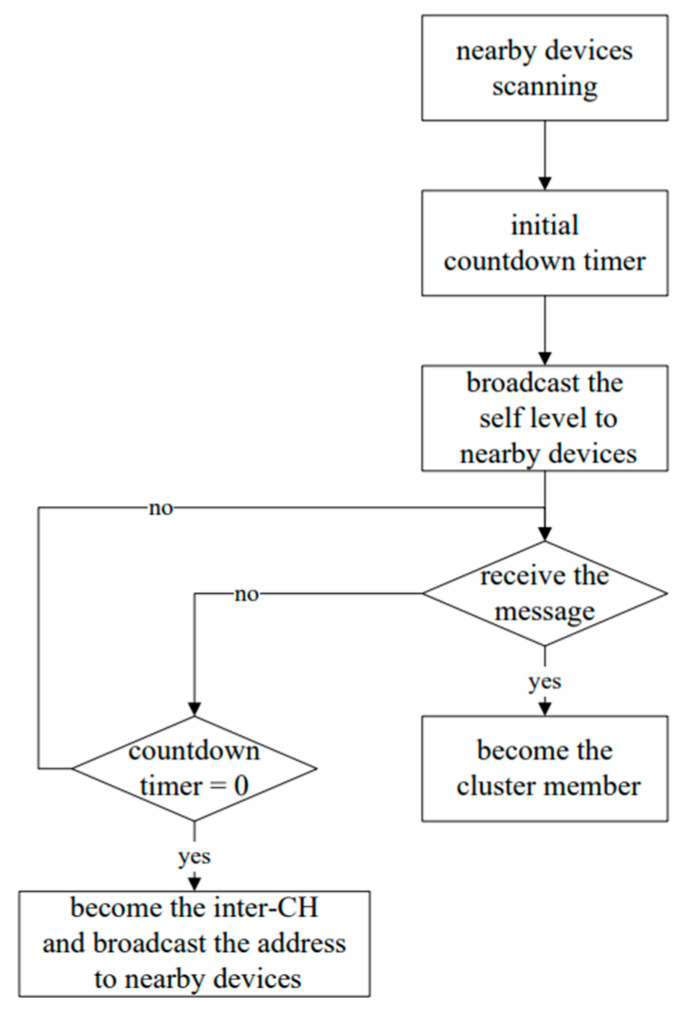
The procedures of the clustering phase.

Without loss of generality, Figure 5 (right) shows four zones with different priority levels for characterizing the topological behavior of the proposed system. The upper right and lower left zones are general wards, where the people in these areas are with priority levels 2 or 3; the people in the upper left zone are all with priority level 3; the lower right zone is the most urgent area, where the people in this area are with priority levels 1 or 2. Moreover, a CH with a different color represents a different priority level. Denote the stars, triangles, and squares as the CHs with priority levels of Level-1, Level-2, and Level-3, respectively, for transmitting the sensing data. The green dots are fixed relays located in each zone, and the cyan-blue dot in the middle is the base station. Figure 6 illustrates the flow diagram of clustering procedures.

#### 3.3.2. Phase 2: Scheduling Algorithm for CHs and Inter-CHs

Considering a scenario with three priority weights, an adaptive transmission scheme is developed to ensure reliable collection of the medical information. Three levels of priority for each patient are given by

- Level 1: Intensive Care Unit S_1_ (e.g., weight of S_1_, $w_{S_{1}}$= 3)
- Level 2: General ward S_2_ (e.g., weight of S_2_, $w_{S_{2}}$= 2)
- Level 3: Normal people S_3_ (e.g., weight of S_3_, $w_{S_{3}}$= 1)

**Figure 7:**
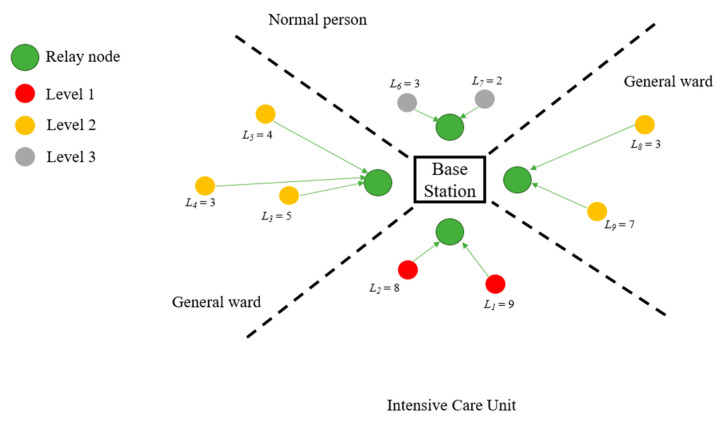
An example of weight calculation given the network topology in Figure 5 (left).

Therefore, for inter-CH *i*, total weights $L_{i}$ can be obtained through the weight equation to decide the transmission priority of the data.

(1) $$L_{i}=N_{CH(S_{1})}^{\left(i\right)}\times 3+N_{CH(S_{2})}^{\left(i\right)}\times 2+N_{CH(S_{3})}^{\left(i\right)}\times 1,$$

where $N_{CH(S_{j})}^{\left(i\right)}$ represents the number of CHs associated with level *j* and inter-CH *i*. Figure 7 shows the relationship between inter-CHs and the corresponding relay after applying the weight equation.

**Figure 8:**
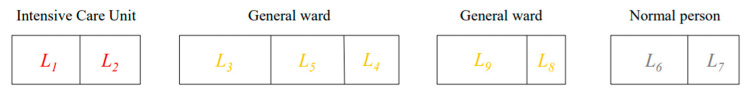
Inter-CHs scheduling.

Referring to Figure 7 with weight calculation, the transmission order of the inter-CHs base on TDMA protocol [43] is described in Figure 8 for communicating with their respective relay nodes. Note that the computation of total weight considers the number of inter-CHs and the priority levels of CHs (e.g., for $L_{1}$ in the Intensive Care Unit, there were three level 2 clusterheads and a level 1 CH in this inter-cluster. Based on the weight equation, we obtain $L_{1}$ = 9. Since there are two inter-clusters in the Intensive Care Unit, two time slots are arranged to transmit the data to the relay node. As the result of each inter-CH’s weight, the transmission time duration is divided into two different time slots, as depicted in Figure 8.

#### 3.3.3. Phase 3: Scheduling Algorithm for Inter-CH and Relay

**Figure 9:**
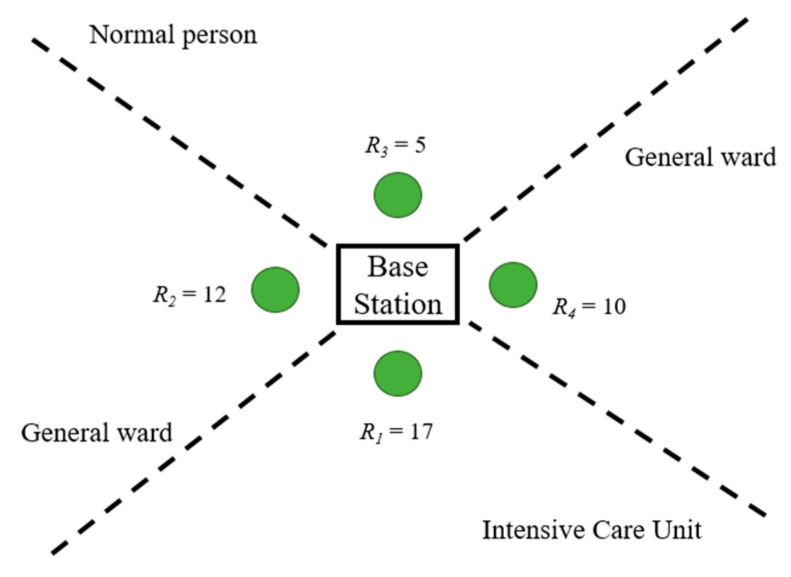
Network clustering and inter-CH selection.

**Figure 10:**
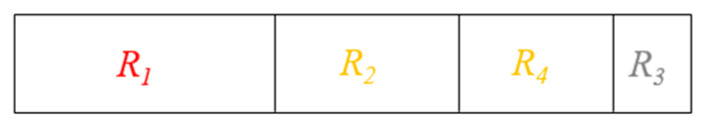
Relay scheduling.

Based on the operations of inter-CHs in Phase 2, this subsection briefly describes a conceptual design of relay scheduling for the multi-relay case. Considering the priority for each relay node, the approach described in Phase 2 can also be applied to schedule the relays. For instance, referring to Figure 7, there are two inter-CHs in Intensive Care Unit. Therefore, the weight of relay *i*, $w_{R_{1}}$, is equal to the summation of the corresponding $L_{i}$ (e.g., $w_{R_{1}}$ = $L_{1}$ + $L_{2}$ = 17). The weights of relay for four wards are shown in Figure 9. Accordingly, weight calculation can be applied to decide the transmission priority by adding the priority levels of its corresponding inter-CHs, as shown in Figure 10.

Based on the operations in Phases 2 and 3 and considering the weights of signals, TDMA protocol assigns the transmission slots among the base station and relay nodes, which is used to schedule the data to transmit from CHs to inter-CHs and from inter-CHs to relay. The setup phase and the steady state phase in the TDMA schedule has been proposed in [13]. Every slot may have a different size, which may depend on the parameter $w_{R_{i}}$, which means if a relay is associated with inter-clusters with high priority or many members in the inter-cluster, the time slot duration will be adaptively adjusted. Moreover, for inter-cluster scheduling, members with higher priority levels are allowed to transmit their data more frequently for monitoring the high-risk patients. As shown in Figure 9, ∑*R_i_* = 44, then we allocate the corresponding size of the time slot for each relay (e.g., the percentage of time slot for *R*_1_ is about 38.64% (17/44) of the transmission time). Accordingly, the scheduling method sets the high-level CH to transmit more frequently, and the inter-CH will fusion the whole data then transmit to relay.

### 3.4. Algorithms for Transmission Scheduling of Physiological Signals

Scheduling packets at sensor node are significantly important for prioritizing applications of WSNs. The nodes collect different types of physiological signals, which have priority to transmit because not all the physiological signals need real-time monitoring so that widely managing the collected signals is essential. Meanwhile, with the constant expanding of network devices, it is impractical to increase the number of queues and network communication resources. Hence, queue scheduling algorithms can be applied to prevent the issue of increased delay and long waiting time during network transmission, which can enhance network transmission efficiency and balance the allocation of network communication resources. This section develops several scheduling algorithms using three physiological signals with different priority levels.

#### 3.4.1. Method 1: Priority Queueing

Each element in the priority queue (PQ) has its own priority, and the higher-priority queue has absolute priority over the lower-priority queue. Packets with a lower priority can be delivered only after the packets of a higher priority are transmitted, and if they have the same priority, they are served by the order of their arrival. This queuing mechanism ensures the delivery of high-priority packets in the event of network congestion. PQ is an unquantified Differentiated services (DiffServ) that only defines pre-defined high-priority packets with priority forwarding rights, but its potential drawback is that a high percentage of priority traffic will cause ordinary traffic to suffer extreme performance degradation, which may make lower priority tasks never be allocated with resources, resulting in a starvation phenomenon.

**Figure 11:**
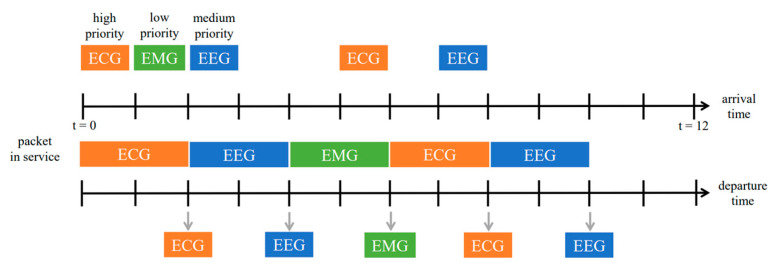
A typical example of priority queueing.

The three physiological signals we used are assigned their own priorities. Since ECG is a vital signal that can be used to identify many signs of disease, ECG is given the highest priority. Consequently, EEG and EMG are the medium and the lowest priority, respectively. As shown in Figure 11, the received physiological signals are placed in a queue for transmission according to their priorities, but when a higher priority signal is added during the waiting period, the lower priority signal can be transmitted only after the higher priority packet has been transmitted. If the sampling rate is greater than the transmission rate, the low-priority physiological signals may have a long waiting time or even fail to be transmitted.

#### 3.4.2. Method 2: WFQ with Round Robin (WFQ_RR)

**Figure 12:**
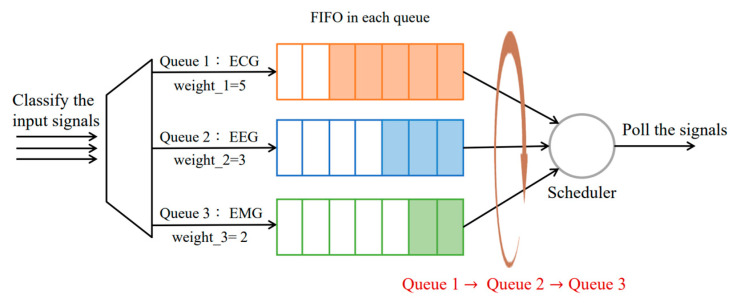
The procedure of the WFQ with Round Robin.

Weighted Fair Queueing (WFQ) is an extension of Fair Queueing (FQ), which shares the link’s capacity in equal subparts, so that different queues are given a fair chance to be scheduled, and to balance the delay and jitter of each flow in general. Compared with FQ, WFQ allows schedulers to specify which fraction of the capacity will be given to each flow by adding a priority consideration in the calculation of packet scheduling. Each queue follows the First in First out (FIFO) principle and is sent according to the order of joining the queue. At the time of queueing, WFQ allocates the bandwidth of each flow according to the weight of the flow, which ensures fairness between the flow of the same priority and expresses the weighting between the flows of different priorities. Last, polling each queue and the corresponding number of packets are sent from the queue according to the bandwidth calculation as follows:

(2) $${BW}_{j}=\frac{w_{j}}{\sum_{i=0}^{N}w_{i}}R$$

where *N* is the total number of queues, *R* is the bandwidth of link, and $w_{i}$ is the weight of each queue, the queue of number *j* will achieve the corresponding bandwidth ${BW}_{j}$ with the calculation. As shown in Figure 12, the physiological signals are placed in their corresponding queues according to their types, where the weight of each queue is 5, 3, and 2, respectively. The bandwidth is allocated after configuring the weight Equation (2) of each queue (i.e., the bandwidth of ECG, ${BW}_{1}=5/10\;R$), and then the packets in the same queue are discarded in order.

#### 3.4.3. Method 3: WFQ with Round Robin and Empty Buffer (WFQ_RR_Empty)

**Figure 13:**
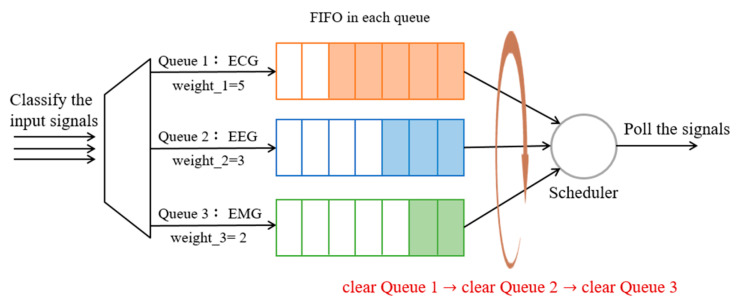
The procedure of the WFQ with Round Robin and empty buffer.

Based on Method 2, each queue of WFQ follows the FIFO principle and the bandwidth is assigned according to the weight of the flow. Although the priority consideration allows higher priority packets to be served first, and the weight assignment enables a fair scheduling opportunity among queues. However, in order to reduce the interaction and the number of rounds between flows, as well as to obtain more important signals at one time, after the bandwidth is allocated according to the weight, the highest priority queue will be clear first before it is rounded to the next queue. As shown in Figure 13, each physiological signal is placed in its corresponding queue, where the bandwidth is allocated after configuring Equation (2) of each queue. Thus, the highest-priority ECG signal will be transmitted first until the queue is empty, and then the second-priority queue is consulted.

#### 3.4.4. Method 4: WFQ with Timestamp and Empty Buffer (WFQ_Timestamp_Empty)

**Figure 14:**
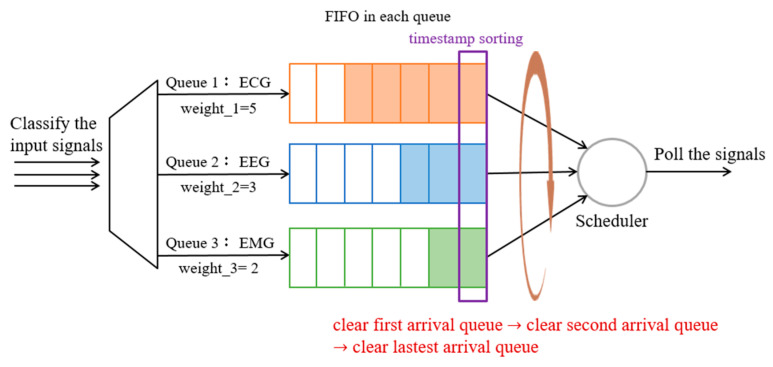
The procedure of the WFQ with timestamp and empty buffer.

Referring to Methods 2 and 3, the higher-priority packets can be served first, but since the sampling frequencies of various physiological signals are different, polling according to the priority order may result in a larger delay for lower priority signals. Accordingly, Method 4 considers the arrival timestamp of the first packet in each queue, then arranges the transmission order according to the timestamp (i.e., the first arriving queue will be transmitted first). As shown in Figure 14, once the signals of different sampling frequencies enter the corresponding queue, the first packet in the current queue generates a timestamp, (e.g., the system timestamp of ECG = 1,662,219,425.096878 (s), EEG = 1,662,219,425.099889 (s) and EMG = 1,662,219,425.072183 (s), respectively). Then, the queue transmission order is determined by comparing the respective timestamp. In the above example, the EMG timestamp is the smallest, which will be transmitted first, followed by ECG, EEG. Therefore, each queue is assigned a transmission bandwidth according to its Equation (2), where each queue still follows the FIFO principle that the packets in each queue are sent in sequence, and then the queue is empty before the next queue is allowed to transmit.

#### 3.4.5. Method 5: WFQ with Timestamp, Priority, and Empty Buffer (WFQ_Timestamp_Priority_Empty)

**Figure 15:**
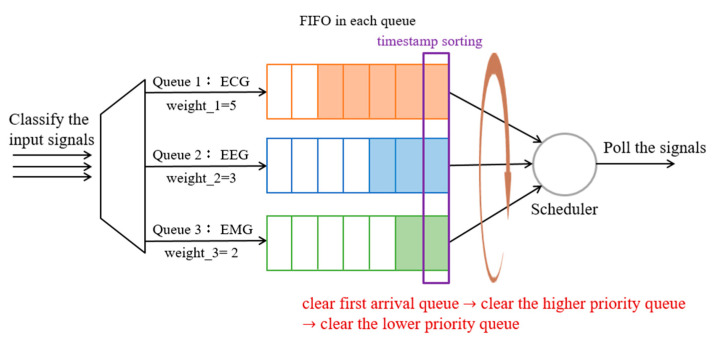
WFQ_Timestamp_Priority_Empty.

On the basis of method 4, the timestamp consideration can provide a possibility for lower priority signals to be allocated resources to reduce latency, higher priority signals still have the necessity to be sent first. For that reason, after obtaining the first timestamp in each queue, the first arriving queue will be served first, and the remaining queues will be sent in order of priority. The transmission bandwidth is allocated according to Equation (2), and the queue will only be polled to the next queue when the queue is empty. As shown in Figure 15, the timestamp from the first packet in each queue is obtained (e.g., the timestamp of ECG = 1,662,219,425.096878 (s), EEG = 1,662,219,425.099889 (s), and EMG = 1,662,219,425.072183 (s), respectively). The EMG with the smallest timestamp will be served first, then the priority of the rest queues will be evaluated. The higher-priority queue ECG will be sent first, and the EEG will be sent only after the queue is cleared. Each queue still follows the FIFO principle that the packets in each queue are transmitted in order.

#### 3.4.6. Method 6: WFQ with Timestamp and Priority (WFQ_Timestamp_Priority)

**Figure 16:**
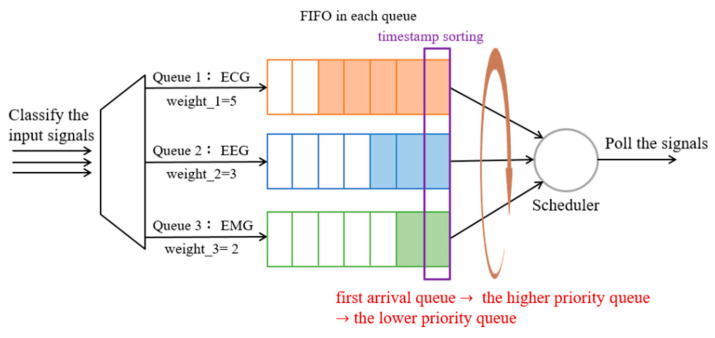
WFQ_Timestamp_Priority.

The timestamp is provided so that the first arriving signal, even if it is low priority, can be delivered first. However, considering that high-priority signals still have the requirement for timeliness, the priority ranking is still necessary. Therefore, after getting the timestamp of the current first packet in each queue, the first arriving queue will be served first, and the remaining queues will be sent in order of priority. While clearing the queue can reduce the number of polling, the waiting time may become longer for higher priority packets. And so, the transmission bandwidth is assigned with the Equation (2), the queue is polled to the next queue after transmission, and the first packet in the queue is dropped first according to the FIFO principle. As shown in Figure 16, comparing the timestamp of the current first packet in each queue (e.g., the timestamp of ECG = 1,662,219,425.096878 (s), EEG = 1,662,219,425.099889 (s), and EMG = 1,662,219,425.072183 (s), respectively). The first arriving queue EMG will be served first, and then considering the priority of the remaining queues, the higher priority queue ECG will be delivered according to the allocated bandwidth before the EEG is forwarded.

## 4. Implementation and Performance Evaluation

### 4.1. Experimental Settings

**Figure 17:**
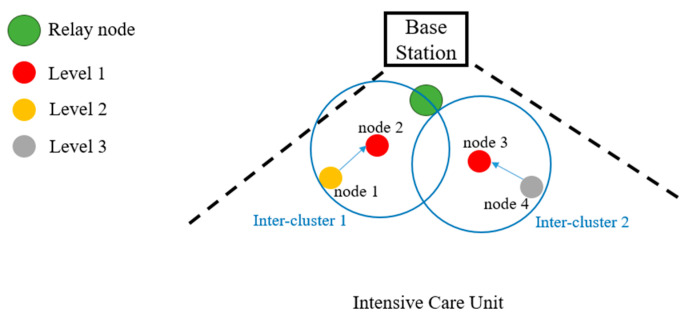
A scenario of experiments.

The experimental field is a single ward, which contains four nodes and a relay node. Through the cluster approach mentioned in Section 3.3.1, the nodes are grouped into two clusters, as shown in Figure 17, where inter-cluster 1 contains two nodes with level-1 and level-2, respectively. And there are two nodes with level-2 and level-3 in inter-cluster 2. Each node schedules the physiological signals according to the method mentioned in Section 3.4 and the direction of the transmission flow is from the sensor nodes to the base station via CHs, inter-CHs, and relays. Considering the strengths of the raspberry pi single-board computer (e.g., its small size, powerful expansion capabilities, and multiple general-purpose input/output pins, supporting Bluetooth and Wi-Fi wireless communication), in this work we use the raspberry pi as a CH or an inter-CH for collecting physiological signals and performing data compression. Moreover, for large amounts of data decompression and analysis, we choose Jetson Nano as a relay node for edge-based applications.

During a network initialization phase, each CH with raspberry pi scans the device to estimate how many adjacent CHs it has and then broadcasts a message of its scheduling priority (1~3) with a random waiting time. At the beginning, the number of inter-cluster members is zero. Each CH updates their neighbor information (i.e., a counter specifying how many neighbors it has detected) and decrease the random waiting time based on each new priority message received. Note that the nodes that hear many neighboring CHs are good candidates for initiating a new inter-cluster; those with few neighbors should choose to wait. When the timer of a CH expires, the CH becomes an inter-CH and broadcasts an address to its neighboring CHs.

**Figure 18:**
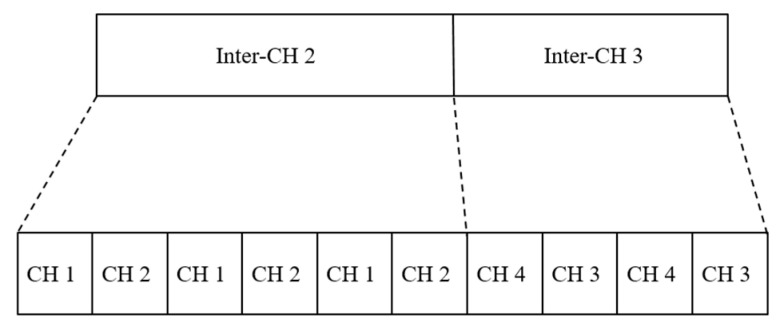
The conceptual communication cycles and transmission slots from Figure 17.

In contrast, when receiving a message with the inter-CH address, the CH joins the inter-cluster and becomes an inter-cluster member. Figure 18 shows the conceptual communication cycles and transmission slots in the proposed network architecture. As the clustering scheme mentioned in Section 3.3, a communication cycle assigns transmission slots among the base station and relay nodes. TDMA is used to schedule the data to transmit from CHs to inter-CHs and from inter-CHs to relay. The scheduling method is setting the high-level CH to transmit more frequently, and the inter-CH will fusion the whole data then transmit to relay. In Figure 17, inter-cluster 1 with level-1 and level-2 CHs, node 1 (i.e., a CH) in the inter-cluster will transmit the data to node 2 (i.e., an inter-CH). Then, the inter-CH will transmit the fusion data, which includes its data to the relay, as shown in Figure 18.

### 4.2. Compression Results

The method mentioned in Section 3.3.2. is applied to denoise the physiological signal based on Wavelet Transform (WT). In our work, the soft threshold function [36,37] is used for all the physiological signals. The criteria for evaluating the denoising performance is the signal-to-noise ratio (SNR), which yields

(3) $$\mathrm{SNR}=10\;\log_{10}\left(\frac{P_{signal}}{P_{noise}}\right)=10\;\log_{10}\left(\frac{\frac{1}{N}\;\sum_{i=1}^{N}x^{2}\left(i\right)}{\frac{1}{N}\;\sum_{i=1}^{N}{\left(x\left(i\right)-\hat{x}\left(i\right)\right)}^{2}}\right)$$

where $x\left(i\right)$ is the noisy signal, $\hat{x}\left(i\right)$ is the denoised signal, and *N* is the length of the signal. Observe that the average noise levels can be reduced effectively with the noise reduction process, as shown in Table 3.

From Section 3.3.3, DWT is applied for physiological signal compression. The criterion of fidelity is adopted to evaluate the compression methods parameters, compression ratio (CR), and percent root-mean-square difference (PRD).

(4) $$CR=\frac{N_{org}}{N_{comp}}$$

(5) $$PRD=100\cdot \sqrt{\frac{\sum_{n=1}^{N}{\left[x_{org}\left(n\right)-x_{r}\left(n\right)\right]}^{2}}{\sum_{n=1}^{N}{\left[x_{org}\left(n\right)\right]}^{2}}}$$

where $N_{org}$ is the number of bits in the original signal, $N_{comp}$ is the number of bits in the compressed signal, $x_{org}$ is the original signal, and $x_{r}$ is the reconstructed signal after compression. Note that the PRD is used to measure the difference between the original signal and the reconstructed signal. The compression result of physiological signals is shown in Table 4.

### 4.3. Analysis on Experimental Results

#### 4.3.1. Performance Comparison of Scheduling Algorithms

In a queue, while packets are waiting to be transmitted on the link, the time cost of waiting for other packets that arrive first to complete their transmission is queuing delay. The queuing delay of a packet will depend on the number of packets that have arrived first or are waiting for transmission to the link. In this experiment, we use three physiological signals as inputs, ECG, EEG, and EMG, and the link bandwidth is 250 Kbps.

**Figure 19:**
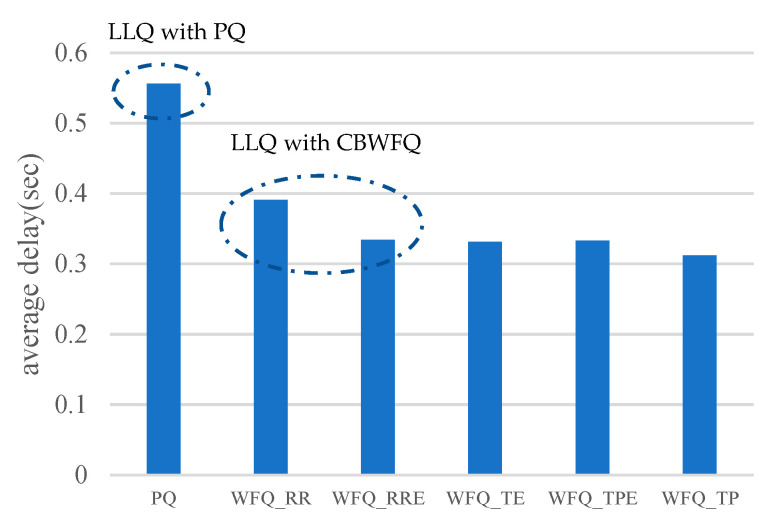
Average delay comparison of physiological signals scheduling.

To assess the proposed algorithms, Figure 19 shows the comparison of the Low Latency Queueing (LLQ) algorithm [21] and the proposed schemes, which describes the average time in the queueing process at the CH for different scheduling algorithms. In a PQ scheme (i.e., LLQ with PQ [21]), if higher-priority packets are inserted frequently, lower-priority packets are probably not allocated with resources, resulting in long queueing delays. For WFQ_RR and WFQ_RRE scheduling schemes, which are related to LLQ with CBWFQ [21], the transmission bandwidth is assigned according to the weight, so that each queue has a fair chance to transmit. However, the lower-priority packets still have to wait before the higher priority queue completes the transmission, so the waiting time is slightly longer. For the proposed WFQ_TE, WFQ_TPE, and WFQ_TP scheduling schemes, in addition to the priority ranking, time stamp is considered to allow the packets to be transmitted even if they are of lower priority but enter the queue first, which results in a shorter waiting time than those with the priority ranking only. As shown in Figure 19, compared to the LLQ with PQ and LLQ with CBWFQ algorithms [21], the performances of the proposed WFQ_TE, WFQ_TPE, and WFQ_TP scheduling schemes are superior or competitive in term of queueing delay.

In order to evaluate the effectiveness of each algorithm in relieving transmission jitter, the average standard deviation (ASD) of the service delay is used as an evaluation index [25]:

(6) $$A_{SD}=\frac{1}{N_{d}}\sum_{i=1}^{K}\sqrt{\frac{1}{N}\sum_{j=1}^{N}{\left(t_{ij}-\bar{t_{i}}\right)}^{2}}$$

where $N_{d}$ is the total number of packet levels, which is 3, K is the packet level,$\;t_{ij}$ is the transmission delay of the *j*th data packet of the *i*th priority level, and $\bar{t_{i}}$ is the average transmission delay of the *i*th level packet. In purpose of obtaining accurate service delay performance, the monitoring time is set to 1 s~15 s [25]. Table 5 shows the average standard deviation of service delay with varying monitoring times. Comparing to the standard deviations in the service delay of WFQ-based algorithms, the PQ algorithm has the largest ASD. As the monitoring time further increases, the service traffic also increases and the ASD of each algorithm shows an increasing trend, but the ASD of the WFQ algorithm is growing at the slowest rate. Observe that the WFQ_TP algorithm has better results than other algorithms in reducing the transmission jitter.

Regarding the delay and jitter of WFQ-based scheduling approaches, Table 6 shows the average delay and average standard deviation of service delay of the LLQ [21] and the proposed WFQ-based algorithms. Observe that comparing to the jitter of LLQ with PQ [21] within 1 s time interval, LLQ with CBWFQ [21], WFQ_TE, WFQ_TPE, and WFQ_TP algorithms achieve 17.07%, 19.51%, 17.07%, and 21.95% lower transmission jitters, respectively. Similarly, compared to the delay of LLQ with PQ [21], LLQ with CBWFQ [21], WFQ_TE, WFQ_TPE, and WFQ_TP algorithms achieve 29.68%, 40.47%, 40.11%, and 43.88% fewer delays, respectively.

#### 4.3.2. The Average Communication Latency

**Figure 20:**
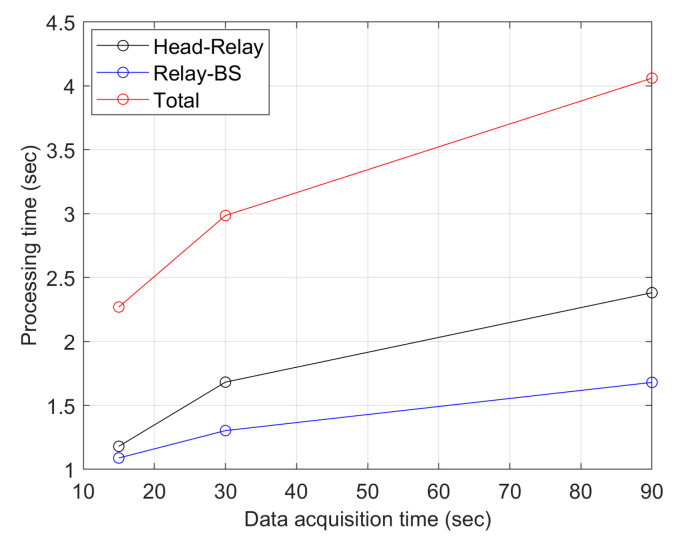
Average delay of the communication.

Referring to the proposed network architecture for communication cycles and transmission time slots in Figure 18, each node compresses physiological signals and schedules the transmission to the CH. Then, TDMA is utilized to schedule the data transmission from the CH to the inter-CH and from inter-CH to relay. The signal sending rate is related to the priority level, which implies the scheduling arrangement of the sending duration. Observe that the time slot durations of inter-CHs are allocated based on priority weightings. Notice that the clustering scheme coordinates the transmission order, which may reduce the delay of data collection. In the case of 15 s of monitoring, one CH will have 6350 bytes of data, and the transmission rate is 150 kbps. The distance between the inter-CH and the CH is 2 m, and the distance between the inter-CH and relay is 1 m. As shown in Figure 20, the longer the monitoring time, the more data will be generated, thus resulting in a longer delay.

#### 4.3.3. Energy Consumption

This set of experiments explores and compares the energy consumption between the proposed clustering scheme and the direct path scheme (i.e., sensed data are forwarded directly to BS in the single-hop communication). Referring to the energy model in [44], the energy consumptions of transmission and reception are:

(7) $$E_{T_{x}}=\left\{\begin{array}{c} kE_{elec}+k\varepsilon_{fs}d^{2},\;d\leq d_{0}\\ kE_{elec}+k\varepsilon_{mp}d^{4},\;d>d_{0} \end{array}\right.$$

(8) $$E_{R_{x}}=kE_{elec}$$

(9) $$E_{Fusion}=M\cdot E_{elec}$$

where $E_{elec}$ is the energy consumed for sending one bit, and $\varepsilon_{fs}$ and $\varepsilon_{mp}$ are, respectively, the amplification coefficients in free space and multipath environments. Given $E_{elec}=50\;\mu J/\mathrm{bit}$ [3.5 mA (on the idle state of the raspberry pi) × 5V × 29 μ (s/bit)], $\varepsilon_{fs}=100\;pJ/m^{2}$, $\varepsilon_{mp}=0.0013\;pJ/m^{4}$, $d_{0}=\sqrt{\varepsilon_{fs}/\varepsilon_{ms}}$, the packet size k = 6350 bytes. Given that the distance between the inter-CH and the CH is two meters and the distance between the inter-CH and relay is one meter, the following results show that the proposed clustering scheme achieves lower energy consumption compared with the direct path scheme (Table 7). As the monitoring time of physiological signals increases, the energy consumption of the transmission also increases. Compared to transmitting all nodes directly to a relay node, the clustering scheme can be more effective in reducing transmission delay and energy consumption for fields with a large number of nodes or a larger amount of data.

In this work, we investigate the energy consumption of the proposed topology scheme with compressed data, comparing to that of the direct path scenario, as shown in Table 7. Moreover, according to the energy saved by compressing data and the energy consumption for running data compression algorithms in edge devices, the net energy income obtained by running an algorithm to compress given data is further examined. Referring to the analysis in [45], we have

(10) $$E_{net}=E_{uncmp}^{tx}-E_{cmp}^{total}=E_{uncmp}^{tx}-(E_{cmp}^{tx}{+E}_{cmp}^{alg})=\left(CR-1\right)E_{cmp}^{tx}-E_{cmp}^{alg}$$

where $E_{net}$ is the net energy income obtained by running a data compression algorithm, which is the difference between $E_{uncmp}^{tx}$ and $E_{cmp}^{total}$, $E_{uncmp}^{tx}$ is the energy consumption for transmitting the raw data, $E_{cmp}^{total}$ is the total energy consumption for compressing data $E_{cmp}^{alg}$ and transmitting the compressed data $E_{cmp}^{tx}$, and CR is the compression rate as described in Equation (4). In the future, we plan to investigate the necessary condition of energy saving, considering the tradeoff among the compression rate CR, the energy consumption for communication and algorithm execution, such that energy conservation can be achieved.

## 5. Conclusions

This paper simultaneously considers signal prioritization and transmission scheduling through the hierarchical clustering method to organize sensors by setting weights and applying data compression techniques at the network edge, which reduces the amount of data transmission. The proposed scheduling algorithm assigns weights according to the physiological signal types and schedules the data to allocate the corresponding transmission bandwidth for ensuring the priority service of critical data. Through the experimental results and evaluation of the existing scheduling mechanisms, the proposed network topology management scheme is shown to achieve the goal of self-organized population health monitoring for IoMT applications. In future work, we are planning to apply the proposed IoMT architecture to possible applications for group health monitoring (e.g., disaster rescue task, fall detection system, or crowd supervision for a group of hikers), determine an efficient tradeoff between compression and data collection to improve the effectiveness of the system design, tackle data security issues in data processing, and further integrate the data of key physiological signals with the relationship between the lesion site and severity for establishing a more comprehensive prioritization scheme.

## Figures and Tables

**Table 1:** A comparative analysis of scheduling mechanisms.

Queueing Algorithms	PQ	RR	WFQ	CBWFQ	LLQ	Proposed
Priority	Yes	No	No	No	Yes	Yes
Bandwidth allocation	No	No	Yes	Yes	Yes	Yes
Classifying based on flow	No	No	Yes	Yes	Yes	Yes
Time in queue	High	Moderate	Moderate	Moderate	Low	Low
Delay sensitive traffic	Yes	No	No	No	Yes	Yes

**Table 2:** List of major features, strengths, and weaknesses of the relevant scheduling schemes.

Scheduling Algorithms	Traffic Type	Features and Strengths	Weaknesses
PQ [22] LLQ-PQ [21]	Real-time	Delay sensitive for high-priority data	The low-priority data are not considered.
RR [23]	Real-time and non-real-time	Different traffic streams get an equal opportunity to be served	The average waiting time is often long.
WFQ [23]	Real-time and non-real-time	Support various packet length, where the sessions with big packets do not receive more scheduling time than the sessions with small packets.	Resource-intensive because of the high resource demands The complex computation of checking the state for each flow and its packets
CBWFQ [22]	Real-time and non-real-time	Utilize traffic classes	Lack of time application support for low delay guarantee
DWRR [26]	Real-time and non-real-time	Handle packets of variable size without knowing the mean size	Services that have a very strict demand on delay and jitter can be affected by the scheduling order of other queues. Offer no priority levels
LLQ-CBWFQ [21]	Real-time	Separate data into different queues to prevent less important data from congesting the network and ensure that critical data are always transmitted promptly.	Need to determine the appropriate priority level for each type of data because low-priority data may be dropped if the network is congested.
GA Methodology [18,19]	Real-time and non-real-time	Explore the energy utilization and task scheduling of the IoMT with the GA evolutionary processing	Selection bias No guarantee of optimal solution
Proposed Algorithm	Real-time and non-real-time	Delay-sensitive for real-time data Decreasing the waiting time of low-priority data. Improved queue management schemes	Resource-intensive because of the computation for checking the state of each flow and its packets

**Table 3:** The result of denoised physiological signals.

Physiological Signals	SNR (dB)
ECG	9.75
EEG	14.71
EMG	17.62

**Table 4:** The result of compressed physiological signals.

Physiological Signals	CR	PRD (%)
ECG	14.76	15.25
EEG	18.97	21.9
EMG	21.65	17.44

**Table 5:** Average standard deviation of service delay.

Monitoring Time (s)	PQ	WFQ_RR	WFQ_RRE	WFQ_TE	WFQ_TPE	WFQ_TP
1	0.041	0.036	0.034	0.033	0.034	0.032
2	0.042	0.035	0.035	0.032	0.033	0.031
3	0.044	0.036	0.035	0.034	0.035	0.033
4	0.042	0.037	0.037	0.035	0.037	0.037
5	0.048	0.040	0.039	0.037	0.038	0.037
6	0.052	0.043	0.038	0.034	0.034	0.036
7	0.048	0.044	0.041	0.039	0.035	0.039
8	0.049	0.041	0.042	0.040	0.041	0.041
9	0.053	0.039	0.043	0.044	0.042	0.041
10	0.054	0.038	0.037	0.043	0.042	0.044
11	0.056	0.042	0.039	0.041	0.038	0.038
12	0.052	0.043	0.044	0.041	0.041	0.040
13	0.051	0.046	0.045	0.042	0.043	0.043
14	0.055	0.045	0.046	0.046	0.044	0.042
15	0.057	0.046	0.045	0.046	0.045	0.044

**Table 6:** Comparison summery of jitter and average service delay.

Algorithms/Features	LLQ with PQ [21]	LLQ with CBWFQ [21]	WFQ_TE	WFQ_TPE	WFQ_TP
Jitter (s)	0.041	0.034	0.033	0.034	0.032
Delay (s)	0.556	0.391	0.331	0.333	0.312

**Table 7:** Energy consumption.

Data Acquisition Time	Clustering Scheme	Direct Path	Reduction
15 s	0.728 J	0.741 J	1.75%
30 s	1.270 J	1.285 J	1.16%
90 s	3.472 J	3.503 J	0.88%

## Data Availability

Not applicable.
